# Motions Add, Orientations Don’t, in the Human Visual System

**DOI:** 10.1371/journal.pone.0075947

**Published:** 2013-10-04

**Authors:** Alan W. Freeman

**Affiliations:** Discipline of Biomedical Science, University of Sydney, Lidcombe, Australia; Harvard Medical School, United States of America

## Abstract

Humans can distinguish between contours of similar orientation, and between directions of visual motion. There is consensus that both of these capabilities depend on selective activation of tuned neural channels. The bandwidths of these tuned channels are estimated here by modelling previously published empirical data. Human subjects were presented with a rapid stream of randomly oriented gratings, or randomly directed motions, and asked to respond when they saw a target stimulus. For the orientation task, subjects were less likely to respond when two preceding orientations were close to the target orientation but differed from each other, presumably due to a failure of summation. For the motion data, by contrast, subjects were more likely to respond when the vector sum of two previous directions was in the target direction. Fitting a cortical signal-processing model to these data showed that the direction bandwidth of motion sensors is about three times the bandwidth of orientation sensors, and that it is the large bandwidth that allows the summation of motion stimuli. The differing bandwidths of orientation and motion sensors presumably equip them for differing tasks, such as orientation discrimination and estimation of heading, respectively.

## Introduction

Orientation selectivity and motion direction sensitivity are two fundamental properties of the mammalian visual system. It is generally accepted that these properties are served by an array of sensors, each tuned to a specific angle along the stimulus axis [1]. Sensor bandwidth, a critical feature of such sensor arrays, has been measured in the orientation domain using techniques such as masking [2], [3]. The results, expressed as full width at half maximum, vary over the range 34°–64°. Estimates of motion direction bandwidth [4]–[6] are also varied, 62°–100°, but have a mean substantially larger than that for orientation. Bandwidths have also been measured from single neurons in the primate: the median orientation bandwidth for a population of primary visual cortical cells was 40° [7], and the mean motion direction bandwidth in area MT ranged from 125° to 147° [8]. Again, motion direction bandwidth substantially exceeds orientation bandwidth.

These findings raise, but do not answer, an important question. Why is motion direction bandwidth larger than orientation bandwidth? One attractive possibility is that motion sensors have to sum over a spread of motion directions, as in the estimation of heading from optic flow [9], [10]. In order to test this hypothesis, the experimental design described here was tailored to measure stimulus summation. A stream of stimuli was delivered and the subject was required to detect a target stimulus. The stream was then analysed to see what stimulus sequences produced a key-press. If the sensor has narrow tuning, stimuli bracketing the target will not add to produce a detection. Finding the largest spread of angles that facilitates a detection should therefore reveal sensor bandwidth.

The experiments were performed both in the laboratory and computationally. The laboratory experiments have been previously published [11], [12]. The computational approach used a multi-stage model for signal processing. Fitting of the model to the empirical data provided not only an estimate of bandwidth, but also illustrated the summation of signals in the motion domain, and the lack of it for the orientation domain.

## Methods

Experimental methods have been previously described [11], [12]; there follows a summary.

### Subjects

Four subjects took part in the orientation experiment and five in the motion experiment. All subjects were aged 19 to 38 and had normal ocular histories, monocular acuities, and binocular disparity thresholds. All subjects but one (an author in [12]) were naïve as to the aims and results of the experiments, and provided written informed consent before taking part. Ethics approval was provided by the University of Sydney Human Research Ethics Committee.

### Stimuli

**Figure 1:**
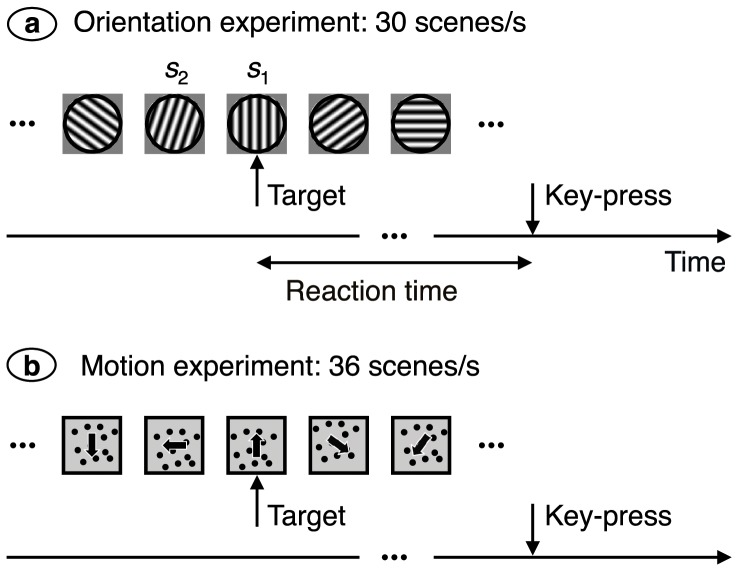
Stimuli. (**a**) In the orientation experiment, randomly oriented gratings were presented in a rapid stream. The subject’s task was to press a key when a target orientation, in this case vertical, was seen. (**b**) For the motion experiment, dots moved coherently in random directions. The subject’s task here was to detect upwards movement.

The stimuli are shown in Figure 1a and 1b. Gratings in the orientation experiment had a spatial frequency of 2 cycles/deg, a contrast of 0.998, and a diameter of 3°. Ten orientations distributed evenly across the range 0°–180° were used. A new orientation was presented 30 times each second, and all ten orientations were equally likely to be selected for a scene. Independent orientation streams were presented to the two eyes, allowing inter-stimulus interactions to be measured both between eyes (an issue of interest in the previously published work) and intraocularly (the issue of interest here). For the motion experiment the stimulus was binocularly congruent. Thirty black dots with a diameter of 0.1° were randomly distributed over a white 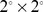 square at the start of a run, and moved as a group thereafter. Two scene rates were used, 36 Hz (two video frames per scene) and 72 Hz (one video frame per scene). Dots were stationary during a scene and generated (apparent) motion as they moved between scenes. The motion had a speed of 3 deg/s and took one of 20 directions distributed evenly across the range 0°–360°: each direction was equally probable for a given scene.

### Procedure

On each (1 minute) run, subjects viewed the stimulus stream and pressed a key when they saw a target stimulus. The target grating for the orientation experiment was horizontal, 45° from horizontal, or vertical, depending on the session. For the motion experiment the target was always vertically upwards.

### Analysis

**Figure 2:**
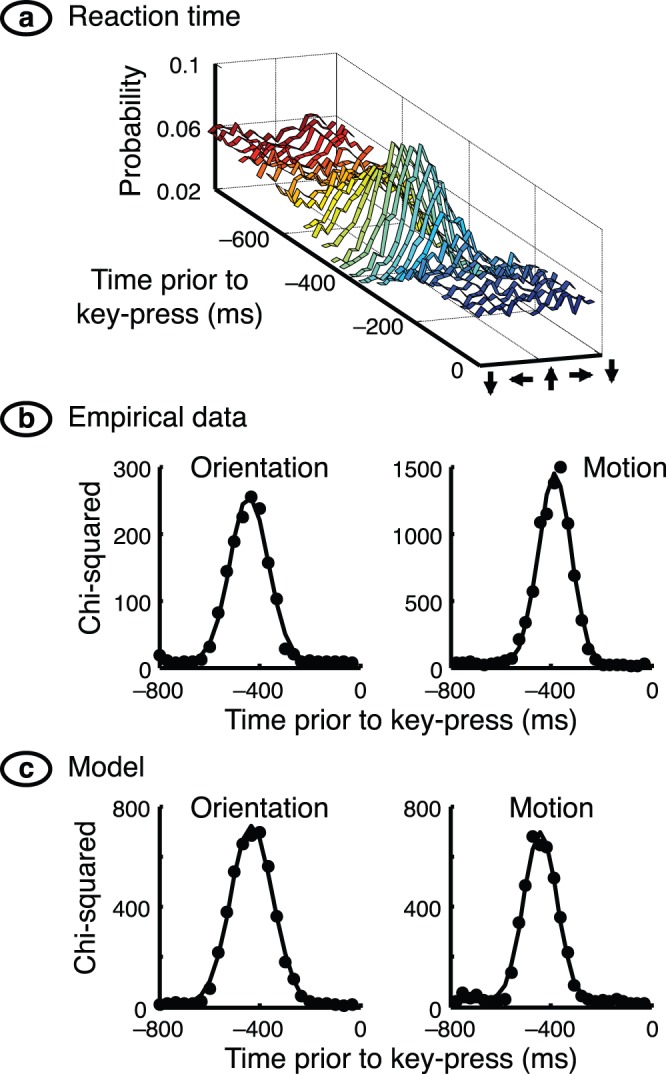
First-order analysis. (**a**) For each key-press the preceding stimulus stream was analysed to find which stimuli were present. The example shown here is for one subject in the motion experiment. The graph provides the probability density of motion direction for each scene prior to a key-press. The densities peak at the target direction for times around 400 ms before the key-press; this interval indicates the reaction time. Densities are close to flat for shorter and longer times. (**b**) Reaction time was quantified by calculating the chi-squared statistic for each density in part a. Chi-squared is shown here as a function of time prior to a key-press. Data from the orientation and motion experiments are shown on the left and right, respectively, and are fitted with a Gaussian function of time. The subject represented at right is the same as for part a of the figure. (**c**) Chi-squared values for the detector model are shown in the same format as for part b of the figure. The peaks are arbitrarily shifted so that reaction times are a little larger than 400 ms.

The stimulus stream preceding each key-press was analysed by finding the stimulus *s*_1_ preceding the key-press by a specified delay, and the stimulus *s*_2_ immediately preceding *s*_1_. The probability density of *s*_1_ peaked at the target value for delays around the reaction time, typically 400 ms, as shown in Figure 2a. A chi-square statistic was calculated for each density by comparing it with a uniform density; see Figure 2b. Densities at all delays were then weighted by their chi-square and summed to collapse them into a single density independent of delay. The resulting densities in the orientation experiment were found to differ little across the three types of target stimulus, and across eye of presentation, and were therefore averaged across these cases.

### Model

**Figure 3:**
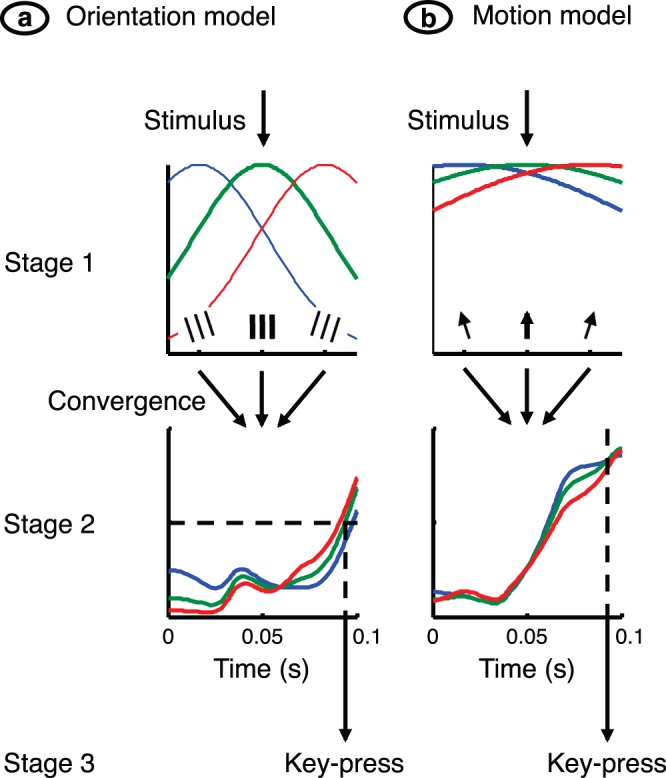
Detector model. (**a**) The orientation model comprised three stages and multiple channels. The first stage consisted of an array of sensors, each tuned to a specific stimulus angle. The second stage comprised an array of channels, each of which received a weighted combination of sensor signals. The second-stage signal was integrated over time, and the third stage triggered a key-press when the signal in the channel tuned to the target stimulus reached a criterion level. (**b**) The motion model was the same as the orientation model except that tuning and weighting functions were wider, and the key-press criterion required that the signal in the channel tuned to the target be higher than all other signals.

The model is an adaptation of a recently published one [13]. It consists of a series of three signal-processing stages, as shown in Figure 3. The first stage represents layer 4 of primary visual cortex, but the location of the latter two stages is undefined. Each of the first two stages has *n* channels, where a channel’s signal represents mean membrane potential across a cortical column. Membrane potential in these two stages is given by
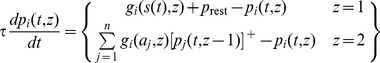



The variables are defined in Table 1, along with their values where appropriate. The stimulus is a step function that changes value at the start of each scene:
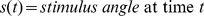
and gain is a Gaussian function of stimulus angle:

**Table 1:** Variables used in equations.

Symbol	Name	Value
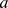	Stimulus angle	
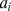	Stimulus angle to which channel *i* is tuned	
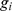	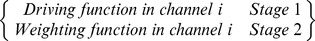	
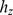	Sensitivity at stage *z*	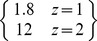
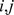	Channel number	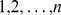
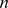	Number of channels	21
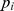	Membrane potential in channel *i*	
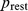	Resting membrane potential in stage 1	–1
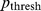	Key-press threshold	1
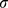	Standard deviation of first-stage tuning and second-stage weighting functions	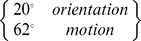
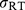	Standard deviation of reaction time	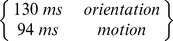
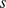	Stimulus	
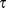	Time constant	32 ms
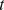	Time	
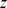	Stage number	1, 2, or 3

Variables are listed along with their symbols and, where appropriate, values.



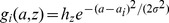



Stage 1 potential is rectified,
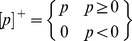
because stage 1 potential is converted to action potential rate before reaching stage 2. Stage 3 implements the decision rule: a key-press is produced when second-stage membrane potential in the channel tuned to the target stimulus exceeds a threshold, *p*_thresh_, or, in the motion experiment, exceeds the potential in all other channels. Key-presses are excluded if they occur closer than 400 ms and key-press times are perturbed by the addition of a Gaussian-distributed random variable with standard deviation of 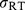. Close examination of Figure 2b shows that the spread of reaction times was wider in the orientation experiment than in the motion experiment. (This difference presumably depends on many factors – such as experimental design, number of trials collected, and pooling of neuronal activity – and is beyond the scope of the present paper.) This empirical finding was replicated for the model data, Figure 2c, by differing settings of 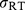 for the two experiments.

The probability of a stimulus pair preceding a key-press, prob(*s*_1_, *s*_2_), was calculated for the model in the same way as for the empirical data. Model parameters were optimised by finding the difference
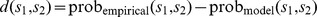
and maximizing



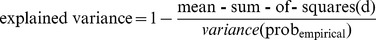



The tuning function’s full width at half maximum is related to 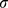 by
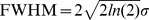



## Results

The empirical data used here come from previously published work [11], [12]. The stimulus was a rapid stream of randomly oriented gratings (Figure 1a) or of randomly directed motions (Figure 1b). The subject’s task was to press a key when a target stimulus (such as a vertical grating or upwards motion) was seen. If two successive stimuli have an angle close enough to the target’s angle, they will both fall within the tuning curve of the sensor tuned to the target. This should lead to summation of the inputs and facilitation of a key-press. Conversely, successive stimuli that differ sufficiently from each other will not sum, with a resultant suppression of key-presses. The width of the tuning curve can therefore be determined by finding the maximum angle between successive stimuli that facilitates key-presses.

**Figure 4:**
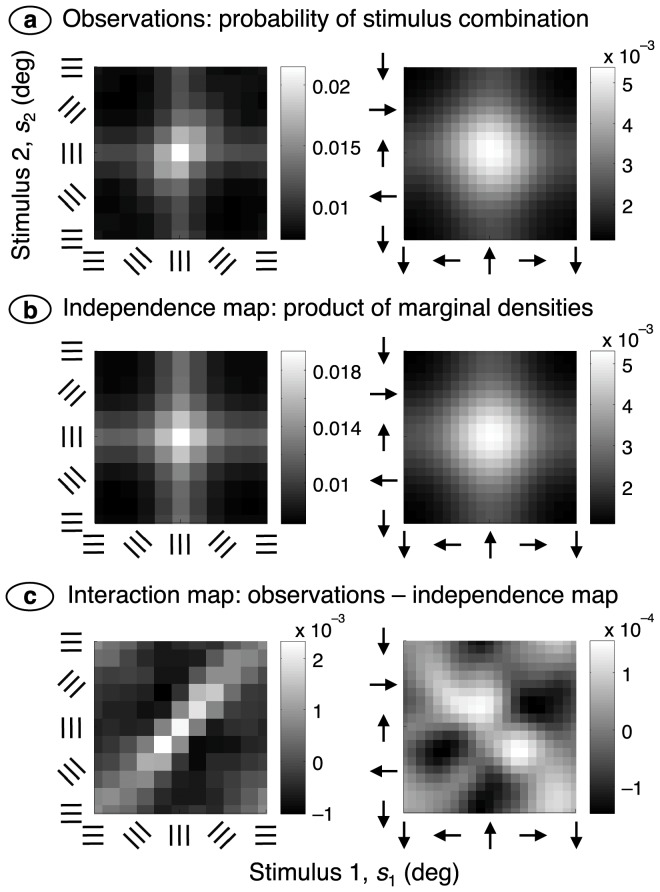
Second-order analysis. (**a**) The horizontal axis shows the stimulus, *s*_1_, that preceded a key-press by the reaction time, and the vertical axis gives the stimulus, *s*_2_, immediately preceding *s*_1_. Grey levels represent the probability of the combination (*s*_1_, *s*_2_), and a guide to grey level is shown in the colour bar. Results from the orientation and motion experiments are shown on the left and right, respectively. (**b**) The independence map was calculated by multiplying the marginal densities in part (a). (**c**) The interaction map shows the difference between the observations (a) and independence map (b). Maps were smoothed using a two-dimensional Gaussian function with a standard deviation of 9° and 27° for the orientation and motion experiments, respectively.

The data were therefore analysed by finding the stimulus, *s*_1_, preceding the key-press by the reaction time and the stimulus, *s*_2_, immediately preceding *s*_1_. Figure 4a shows the proportion of key-presses that occurred at each combination (*s*_1_, *s*_2_) and, not surprisingly, most key-presses occur when both stimuli are close to the target value. This pattern would occur if the two stimuli acted independently to produce a key-press. What we are interested in, by contrast, is the interaction of successive stimuli in producing a key-press. The interaction was obtained in two further steps. First, it was assumed that stimuli *s*_1_ and *s*_2_ act independently in producing a key-press. The resulting pattern (obtained by multiplying the marginal probability densities in Figure 4a) is shown in Figure 4b. Second, the independence pattern was subtracted from the observations to produce the interaction map in Figure 4c. The statistical significance of the interaction was tested by using the key-press counts underlying the observations in Figure 4a. Counts were added across subjects and collapsed into a 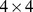 table. A chi-square test on the relationship between the two stimulus variables was significant 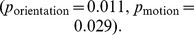


The interaction map differs strikingly between the orientation and motion experiments. In the latter case there is a band of elevated probabilities, that is, facilitation, along the negative diagonal. This indicates that successive stimuli, one on either side of the target stimulus, combine to produce a key-press. This vector summation implies a broad tuning curve that can sum the effect of motion stimuli differing substantially in their directions. For the orientation experiment, on the other hand, the facilitation lies along the positive diagonal, implying that successive gratings must have much the same orientation in order to facilitate a key-press. This, in turn, suggests a narrow tuning curve for orientation.

**Figure 5:**
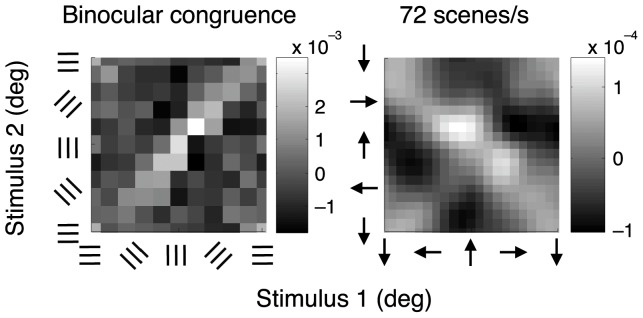
Interaction maps for additional experimental conditions. The orientation experiment was performed for both binocularly incompatible stimuli (Figure 4) and for binocularly congruent stimuli, shown on the left of this figure. The motion experiment was performed with two scene rates, 36 Hz (Figure 4) and 72 Hz, shown here on the right side. The similarity of the interaction maps in this and the previous figure shows the robustness of the results.

Figure 5 shows the robustness of these findings. The interaction map on the left was obtained by presenting the same stream of orientations to each eye (rather than independent streams, as in the previous figure). Facilitation along the positive diagonal is clear but, because a smaller volume of data was collected for the binocularly congruent case, the remainder of the results use the binocularly incongruent data. The right side of Figure 5 shows a motion experiment in which a new motion was presented 72 times each second (rather than 36 Hz as in the previous figure). Facilitation along the negative diagonal is just as pronounced as with the 36 Hz movie. The latter case is used from here on because its scene rate is close to that used in the orientation experiment.

**Figure 6:**
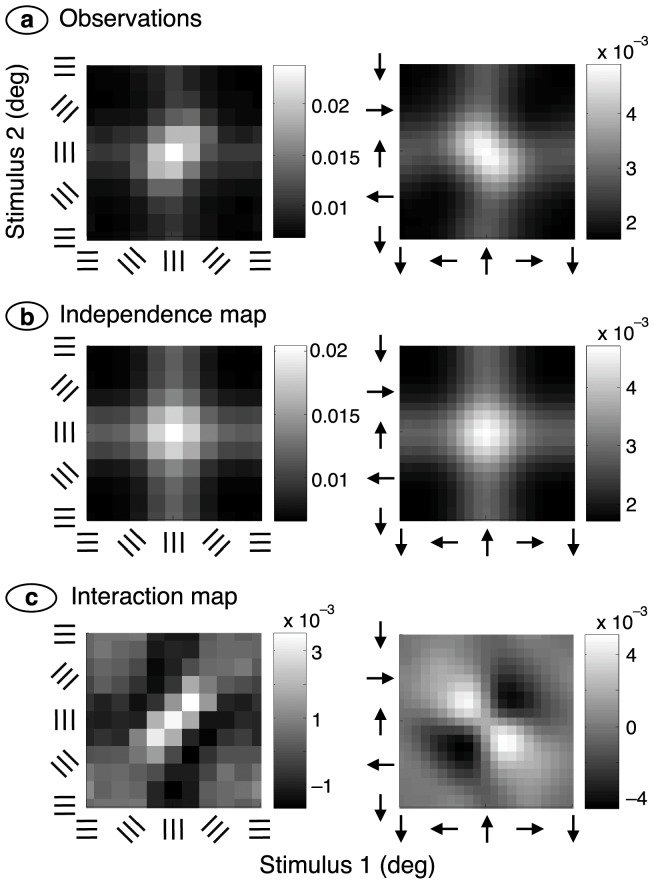
Second-order analysis for the model. (**a**), (**b**), (**c**). Key-presses were analysed in the same way as for the empirical data (see Figure 4). The simulated interaction maps correspond closely with the empirical ones.

A signal-processing model, shown in Figure 3, was used to test the idea that tuning bandwidth accounts for the differing interaction maps. The model, adapted from Hesam Shariati and Freeman [13], has three stages. The first is an array of sensors tuned to differing stimulus angles. Each sensor, which has a resting hyperpolarisation and a rectified output, represents a population of simple cells in a column of primary visual cortex. The second stage sums signals from a range of first-stage channels. The third stage generates a key-press if the channel tuned to the target stimulus reaches a criterion level of activity. Stimuli such as those in Figure 1a and 1b were presented to the model and the resulting key-presses were analysed as with the empirical data. The results, shown in Figure 6, closely reproduce the empirical patterns. The models used to simulate the two experiments differed in two respects. First, the sensor bandwidths for orientation and motion direction were 47° and 146°, respectively. Second, the criterion for generating a key-press in the orientation experiment was that the signal in the second stage exceed a criterion level for the channel tuned to the target. Given that tuning curves were wider in the motion experiment, several neighbouring channels approached this level together. The key-press criterion here, therefore, was that the second-stage signal in the channel tuned to the target exceed that of all other channels.

**Figure 7:**
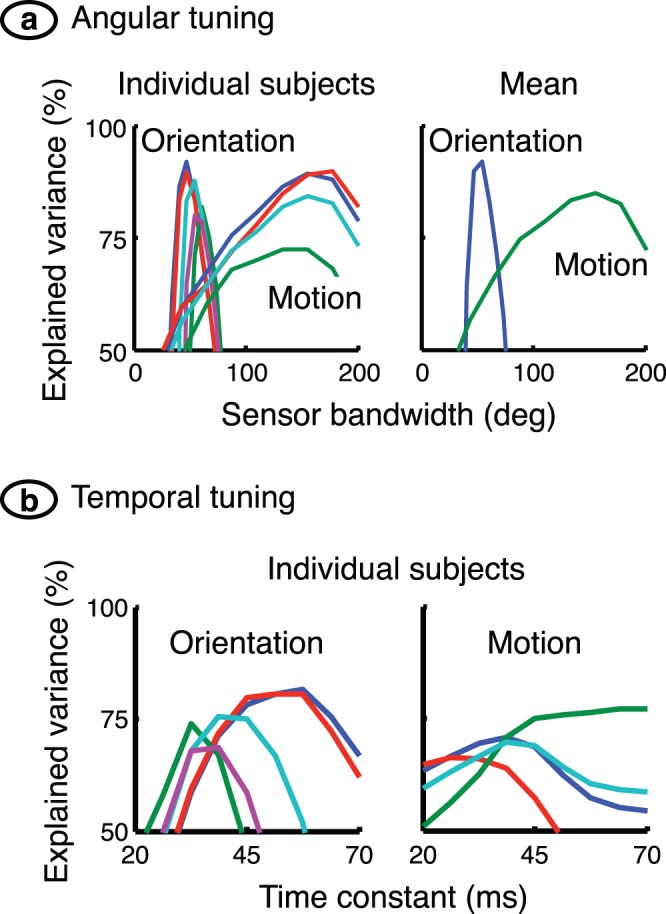
Sensitivity analysis of model parameters. (**a**) The graphs show explained variance, a measure of the match between model and empirical responses, versus the bandwidth of the sensor tuning function. There is one line for each subject on the left, and the means across subjects are shown on the right. The optimal bandwidth for motion sensors is about three times that of orientation sensors. The model explains 92% and 85% of the variance for the orientation and motion experiments, respectively. (**b**) A sensitivity analysis was also performed for model time constant. The results, shown here, produced best fits that differed widely between subjects. There was therefore no value in computing a mean across subjects.

We can now see the mechanism underlying the interaction maps. Facilitation along the negative diagonal in the motion experiment results from summation of motions bracketing the target direction whereas facilitation in the orientation experiment only occurs when successive stimuli activate the same (narrowly-tuned) channel, producing facilitation along the positive diagonal. The bandwidths used in the simulations were obtained by maximising the explained variance. Figure 7, which shows a sensitivity analysis for the optimisation procedure, clearly illustrates the large difference between orientation and motion direction bandwidths. It also shows that the model, which explains at least 85% of the variance, fits the data well.

The perception of brief or fast movements requires rapid visual signal processing. It is possible, therefore, that the differing summation properties of orientation and motion sensors depend on temporal tuning rather than tuning for contour orientation and motion direction. To test this idea, bandwidths in the orientation and motion models were set equal to the value, 68°, at which the curves cross in the right graph of Figure 7a. Time constant was then optimised separately for the two models. The result, shown in Figure 7b, is unacceptable: the solution differs widely between subjects, and explained variance is substantially less than when bandwidth is optimised. The conclusion remains, therefore, that the best way to explain the differing results in the orientation and motion experiments is through small and large bandwidths, respectively.

## Discussion

There were differences in the methods used for the orientation and motion experiments. Before discussing the results, we need to be sure that the difference in bandwidths measured in the two experiments was not due to differing methods. First, both binocularly congruent and incongruent stimuli were used in the orientation experiment, but only congruent stimuli for the motion experiment. A comparison of the left sides of Figures 4c and 5, however, shows that facilitation lies along the positive diagonal for both congruent and incongruent stimulation, implying the same response summation mechanism in these two cases. Second, orientation and motion data were collected at differing scene rates. But the right sides of Figures 4c and 5 show that the interaction maps for the motion experiment were of the same form at 72 Hz and 36 Hz, making it likely that they would also have the same form at 30 Hz, the scene rate for the orientation experiment. Third, the stimulus area was a 3° wide circle for the orientation experiment and a 2° wide square for the motion experiment. Previous work, however, has shown that stimulus detectability changes only slowly with the stimulus area in both the orientation [14] and motion [15] domains.

The bandwidths measured here compare well with values obtained from single-neuron studies in the primate. Quantifying tuning curve bandwidth as full width at half maximum, the orientation bandwidth estimate, 47°, is close to the median value, 40°, for a population of cells in macaque primary visual cortex [7]. The motion bandwidth estimate, 146°, lies within the range of values, 125°–147°, measured in macaque cortical area MT [8]. There is less agreement with previous psychophysical work. Whereas the orientation bandwidth measured here falls within the range of previous estimates [2], [3], the motion bandwidth is substantially larger than those found in previous work [4]–[6]. The reason for this discrepancy probably lies in the methodology. The previous studies used a masking or adapting stimulus that moved in a direction differing from that of the test stimulus. When the angle between the two directions was large, the effect of the mask/adaptor was weak. It has been shown, however, that weak pedestal stimuli improve responses to superimposed test stimuli rather than reducing them [16], [17]. It may have been, therefore, that the masks and adaptors with motion directions far from the tested direction had a facilitatory effect, thereby narrowing the measured motion direction bandwidth.

The model also explains a counter-intuitive aspect of the empirical data. The left side of Figure 4c shows that consecutive stimuli with the same orientation facilitate a key-press even when neither stimulus has the target orientation. This form of facilitation occurs in the model as follows. Consecutive stimuli of the same orientation sum their effects in a single first-stage sensor, producing a suprathreshold signal in that sensor. When the stimulus orientation is close to the target value this signal converges onto the second-stage channel that is tuned to the target, increasing the probability of a key-press. Consecutive orientations that bracket the target, by contrast, produce subthreshold signals in differing sensors and no excitation of second-stage channels. A simpler model, with only a single integrative stage or without rectification of the first-stage signal, could not explain this critical empirical observation.

The Introduction raised the question, why is motion direction bandwidth larger than orientation bandwidth? There are two questions implied here, one about mechanisms and the other about biological advantage. The difference in mechanisms between orientation selectivity and motion sensitivity is probably best understood in terms of stimuli and the simple cell receptive field. Testing orientation selectivity requires a stimulus sufficiently extended to provide at least one contour. The stimulus will typically cover a simple cell receptive field, activating both on- and off-subfields. A suboptimal orientation will then result in destructive interference between on- and off-subfield responses, resulting in a narrow bandwidth. In contrast, motion sensitivity must be tested with stimuli small enough to avoid extended contours in order to prevent the confounding effects of orientation selectivity. The dots typically chosen are smaller than a subfield and therefore produce little or no destructive interference. A broad bandwidth results. Given that neurons in area MT of primate visual cortex inherit key properties from motion-sensitive cells in primary visual cortex [18], MT motion direction bandwidth may also be determined by the simple cell’s properties.

The second question implied in the Introduction is this: what is the biological advantage of having motion direction bandwidths that are substantially larger than orientation bandwidths? The large difference between orientation and motion bandwidths makes intuitive sense. A flock of flying birds and a bush moving in the wind both provide a population of motion vectors, and vector summation should provide a good estimate of overall direction. Similarly, while navigating through the environment, heading can be calculated by adding optic flows on either side of the body. By contrast, there is clear value in being able to discriminate a sharp instrument from a blunt one: averaging the orientations of the contours defining the instrument’s point has no obvious biological advantage.
